# Evaluation of the effect of the new methoxy-stilbenes on expression of receptors and enzymes involved in estrogen synthesis in cancer breast cells

**DOI:** 10.1007/s11010-017-3230-7

**Published:** 2017-11-30

**Authors:** Barbara Licznerska, Hanna Szaefer, Marcin Wierzchowski, Renata Mikstacka, Katarzyna Papierska, Wanda Baer-Dubowska

**Affiliations:** 1 Department of Pharmaceutical Biochemistry, Poznan University of Medical Sciences, Poznań, Poland; 2 Department of Chemical Technology of Drugs, Poznan University of Medical Sciences, Poznań, Poland; 3 Department of Inorganic and Analytical Chemistry, Collegium Medicum, Bydgoszcz Nicolaus Copernicus University, Toruń, Poland

**Keywords:** Methoxy-stilbenes, Breast cancer epithelial cells, ERα, AhR, CYP1A1, CYP1B1

## Abstract

Our previous study showed that the new synthetic methoxy-stilbenes, 3,4,2′-trimethoxy-*trans*-stilbene (3MS), 3,4,2′,4′-tetramethoxy-*trans*-stilbene (4MS), and 3,4,2′,4′,6′-pentamethoxy-*trans*-stilbene (5MS), modulate the constitutive expression of enzymes and receptors involved in estrogen metabolism in breast immortalized epithelial MCF10 cells. In this study, we evaluated the effect of 3MS, 4MS, and 5MS in comparison to resveratrol activity in MCF7 estrogen-dependent and MDA-MB-231 estrogen-independent breast cancer cell lines. 3MS similarly to resveratrol reduced the expression of estrogen receptor α in MCF7 cells. However, in these cells, 5MS reduced the most *CYP19*, the gene encoding aromatase, at mRNA transcript level. In contrast, in the MDA-MB-231 cells, the most efficient inhibitor of *CYP19* expression was 3MS, reducing the level of its protein by ~ 25%. This stilbene also inhibited the aromatase activity in a recombinant protein system with IC_50_ value ~ 85 µM. Treatment with the methoxy-stilbenes reduced the level of estradiol in culture medium. The most significant reduction was exerted by 3MS. None of the tested stilbenes including resveratrol changed significantly the expression of AhR, although CYP1A1 protein level was slightly reduced in MDA-MB-231 cells, while CYP1B1 expression was increased in these cells as a result of treatment with 3MS, but only at the transcript level. Overall, these results show weak or moderate effect of the new methoxy-stilbenes on the expression of key proteins involved in estrogens metabolism in cancer breast cells. However, the reduced *CYP19* expression and activity upon 3MS treatment in metastatic MDA-MB-231 cells require the further studies.

## Introduction

Resveratrol (3,5,4-trihydroxystilbene), a naturally occurring phytoalexin, has been found to possess diversified biological activities including anti-oxidative, anti-inflammatory, and anti-carcinogenic [1]. However, its clinical application is limited because of relatively poor bioavailability due to its fast metabolism [2]. Thus, its analogs showing improved pharmacokinetic parameters and enhanced activity are searched. Comparing to resveratrol, its derivatives with *ortho*-methoxy substituents, such as 3,4,5,4′-tetramethoxy-*trans*-stilbene (DMU-212), have been found to be more potent anti-carcinogenic agents in some in vitro and in vivo studies [3, 4]. Moreover, our earlier data showed that the new synthetic methoxy-stilbenes, 3,4,2′-trimethoxy-*trans*-stilbene (3MS), 3,4,2′,4′-tetramethoxy-*trans*-stilbene (4MS), and 3,4,2′,4′,6′-pentamethoxy-*trans*-stilbene (5MS), modulated the constitutive expression of enzymes and receptors involved in estrogen synthesis and catabolism in breast immortalized epithelial MCF10 cells. These observations indicated that methoxy-stilbenes may affect the estrogens metabolism. Moreover, the number of methoxy groups may play a role in its anti-estrogenic activity [5]. Estrogens and estrogen receptor (ER) signaling pathway play the key role in breast cancer etiology. In ER-dependent mechanism, estrogens may contribute to cancer development acting as promoters by stimulating cell proliferation through the activation of estrogen-ER complex-dependent transcription of specific genes [6].

Distinct ERs genes located on chromosomes 6q25.1 and 14q23.2, encode two ER forms, ERα and ERβ, respectively [7]. ERα is expressed in not more than 10% of normal breast epithelium, but approximately in 50–80% of breast tumors [8]. Moreover, experiments with ER knockout mice demonstrated that ERα is involved in promotion and progression of breast cancer [9].

Estrogens may also exert tumorigenic effects in ER-independent pathway through the reaction of their active metabolites with DNA [10]. The expression of genes metabolizing estrogens such as CYP1A1, CYP1A2, and CYP1B1 is activated by aryl hydrocarbon receptor (AhR).

Several studies have pointed out the inhibition of the ER pathway by AhR in breast cancer as a result of accelerating ER ubiquitination and degradation [11, 12].

Based on this observation, it was suggested that AhR is a tumor suppressor in human breast cancer. In this regard, AhR has been shown to inhibit invasive and metastatic potential of human breast cancer stem-like cells. Moreover, exogenous AhR agonists were able to promote cell differentiation regardless of ER status [13].

On the other hand, some studies have shown that AhR induced intratumoral estrogen synthesis [14, 15]. Although limited information is available on these seemingly opposite effects of AhR in breast cancer, recently presented data indicate that AhR stimulates intratumoral estrogen biosynthesis by inducing aromatase expression in three breast carcinoma cell lines: MCF7, T47D, and MDA-MB-231, resulting in increased cell proliferation. Additionally, results obtained from patients with breast cancer supported this finding [16].

Resveratrol was shown to be a competitive antagonist of classic AhR ligands such as dioxin and an inhibitor of transactivation of dioxin-inducible cytochrome P450 genes [17].

Our earlier study showed reduced expression of *CYP19, AhR* and *CYP1A1* and *CYP1B1* as a result of treatment of the MCF10A breast epithelial cells with 5MS. At the same experimental setting resveratrol increased *CYP19* transcript and protein level [5]. MCF10A cell line represents benign stage of breast cancer development and is considered as a model of carcinogenesis developed through non-ER-mediated pathways [18, 19].

In this study, we evaluated the effect of 3MS, 4MS, and 5MS in comparison to resveratrol in MCF7 estrogen-dependent and MDA-MB-231 estrogen-independent breast cancer cell lines. Although both cell lines derived from human breast adenocarcinoma, the latter is highly metastatic.

## Materials and methods

### Chemicals

Methoxy-stilbenes, 3,4,2′-trimethoxy-*trans*-stilbene (3MS), 3,4,2′,4′-tetramethoxy-*trans*-stilbene (4MS), and 3,4,2′,4′,6′-pentamethoxy-*trans*-stilbene (5MS), were synthesized in the Department of Chemical Technology of Drugs, PUMS, as described previously [5]. Resveratrol, dithiothreitol, antibiotics solution (10^4^ U penicillin, 10 mg streptomycin, 25 μg amphotericin B), bovine serum albumin, dimethylsulfoxide (DMSO), Dulbecco’s Modified Eagle’s Medium (DMEM), 3-(4,5-dimethylthiazol-2-yl)-2,5-diphenyltetrazolium bromide (MTT), RIPA buffer, trypsin, Tris, and tRNA from *E. coli* were purchased from Sigma Chemicals Co. (St. Louis, MO, USA). Primary antibodies against ERα, CYP19, CYP1A1, and CYP1B1 were obtained from Abgent (San Diego, CA, USA). Primary antibodies against AhR, β-actin, and secondary antibodies were supplied by Santa Cruz Biotechnology (Santa Cruz, CA, USA). Protease inhibitor tablets were obtained from Roche Diagnostics GmbH (Penzberg, Germany). SDS-PAGE Gels (7.5, 10, and 12%) and the Western blotting detection system were purchased from Bio-Rad Laboratories (Hercules, CA, USA). All other compounds were readily available commercial products. Resveratrol and its methoxy derivatives were dissolved in DMSO at the concentration of 100 mM and stored at − 20 °C.

### Cell culture and treatment

MCF7 (ATCC^®^ HTB22™) and MDA-MB-231 (ATCC^®^ HTB26™) cell lines were purchased from the European Collection of Cell Cultures (Salisbury, Wiltshire, UK). The cells were cultured in DMEM supplemented with 10% fetal bovine serum and 1% antibiotics solution. To assess the effects of tested compounds, the cells were grown in the presence of 5% fetal bovine serum. Experiments were conducted at a cell density of 70% confluence at standard conditions (5% CO_2_/95% air). After a 24-h preincubation period, the cells were treated with the tested compounds at the doses selected based on viability assay. The incubation was continued for subsequent 72 h. Control cells were treated with vehicle (DMSO). The concentration of DMSO did not exceed 0.1%.

### Cell viability assay

The effect of resveratrol and its methoxy derivatives on cell viability was assessed with the MTT assay, according to standard protocols. The cells were seeded in a 96-well culture plate. After 24-h preincubation period, 1–100 µM of resveratrol or methoxy-stilbenes in the culture medium were added and the cells were incubated for 72 h. Subsequently, the culture medium was removed and a fresh PBS buffer containing MTT salt (0.5 mg/mL) was added to the wells. After a 4-h incubation, the formazan crystals were dissolved in acidic isopropanol and the absorbance was measured at 570 and 690 nm. All of the experiments were repeated three times, with at least three measurements per assay. In all of the subsequent experiments, non-toxic concentrations of methoxy-stilbenes and resveratrol (viability level above 70%) were used, ranging from 0.5 to 5 µM, depending on compound.

### Measurements of ERα, AhR, CYP19, CYP1A1, and 1B1 mRNA transcripts (quantitative real-time PCR)

Total RNA was isolated, using the GeneMATRIX UNIVERSAL DNA/RNA/protein kit (EURx Ltd., Gdańsk, Poland) and the first-strand cDNA was generated from total RNA using the dART RT-PCR kit (EURx Ltd., Gdańsk, Poland) according to manufacturers’ recommendations. Primer pairs capable of hybridization with unique regions of the appropriate gene sequence were designed in Beacon Designer (PREMIER Biosoft Intern.) as described previously [5, 20]. The quantitative real-time PCR was performed in triplicates using SyberGreen on the Chromo4 (Bio-Rad Laboratories, CA, USA) or the LightCycler96 (Roche Diagnostics GmbH, Penzberg, Germany). The final reaction mixture contained 80–250 nM of each primer, 0.5 µL of cDNA, 1 µL of tRNA, and 4 µL of the 5× HOT FIREPolEvaGreen qPCR Mix Plus (Solis BioDyne, Tartu, Estonia), with RNAse-free water up to 20 µL. All reactions were run in triplicate. The protocol started with a 15-min enzyme activation at 95 °C, followed by 40–50 cycles of 95 °C for 15 s; 60 °C for 20 s; 72 °C for 20 s; and the final elongation at 72 °C for 5 min. The melting curve analysis was used for product size verification. Experiments were normalized for the expression of TATA box binding protein (*TBP*). The Pfaffl relative method was used for fold-change quantification.

### Measurements of ERα, AhR, CYP19, CYP1A1, and 1B1 protein levels (Western blot)

Whole cell lysates were prepared using the RIPA buffer. Samples containing 100 µg of proteins were separated on 7.5% (AhR), 10% (CYP19, CYP1A1, CYP1B1, and β-actin) or 12% (ERα) SDS-PAGE gels and transferred to nitrocellulose membranes. After blocking with 10% skimmed milk, the proteins were probed with rabbit polyclonal ERα, goat polyclonal AhR, rabbit polyclonal CYP19, rabbit polyclonal CYP1A1, rabbit polyclonal CYP1B1, and rabbit β-actin antibodies. As the secondary antibodies, the alkaline phosphatase-labeled anti-goat IgG or anti-rabbit IgG were used. Protein contents were measured using albumin as a standard and the β-actin protein as an internal control. The amount of immunoreactive product in each lane was determined by densitometric scanning using a BioRad GS710 Image Densitometer (BioRad Laboratories, Hercules, CA, USA). The values were calculated as relative absorbance units (RQ) per mg protein.

### Quantification of estradiol in culture medium

The concentration of estradiol in culture medium after 72 h of MCF7 cells incubation with methoxy-stilbenes or resveratrol was assessed by an enzymatic immunoassay according to Jin et al. [21] using commercial kit (Estradiol EIA Kit, Cayman Chemical Company, Ann Arbor MI, USA) and following the manufacturer’s instructions. Briefly, the assay was based on the competition between estradiol and an estradiol-acetylcholinesterase conjugate for a limited amount of estradiol antiserum. The antiserum-estradiol and antiserum-estradiol-acetylcholinesterase complexes bound to mouse monoclonal anti-rabbit IgG, previously attached to ELISA microplate. Then substrate for acetylcholinesterase was added, and the absorbance of the yellow product of this enzymatic reaction was measured at 405 nm. The concentration of estradiol in medium samples was expressed in pg/mL, using standard curve on each run. The data from three independent experiments were analyzed.

### Aromatase assay in vitro in human recombinant CYP19

The enzyme activity determinations were performed with the use of CYP19/MFC High Throughput Inhibitor Screening Kit (Gentest, a BD Biosciences Company, Woburn, MA, USA). The methoxy-stillbenes were dissolved in acetonitrile. The effect of test compounds on the aromatase activity was evaluated in the concentration range 0.045–100 µM. The procedure was performed according to the manufacturer’s instruction with the use of 96-well black microtiter plates. After the serial dilution of the test compounds, the samples were preincubated for 10 min with NADPH generating system. Then, CYP19 (7.5 nmols final concentration) and its substrate, 7-methoxy-4-trifluoromethyl coumarin (125 µmols final concentration), were added and the reaction mixture was incubated for 30 min. The reaction was terminated by adding Tris base (0.5 M). The fluorescence of 7-hydroxy-4-trifluoromethyl coumarin was determined at the 409 nm excitation and 530 nm emissions wavelengths. Ketoconazole was used as a positive control. The determinations were performed twice in duplicates.

### Statistical analysis

Statistical analysis was performed by one-way ANOVA. The statistical significance between the experimental groups and their respective controls was assessed by Tukey’s post hoc test, at *P* < 0.05.

## Results

### The effect of resveratrol and methoxy-stilbenes on cells viability

**Fig. 1:**
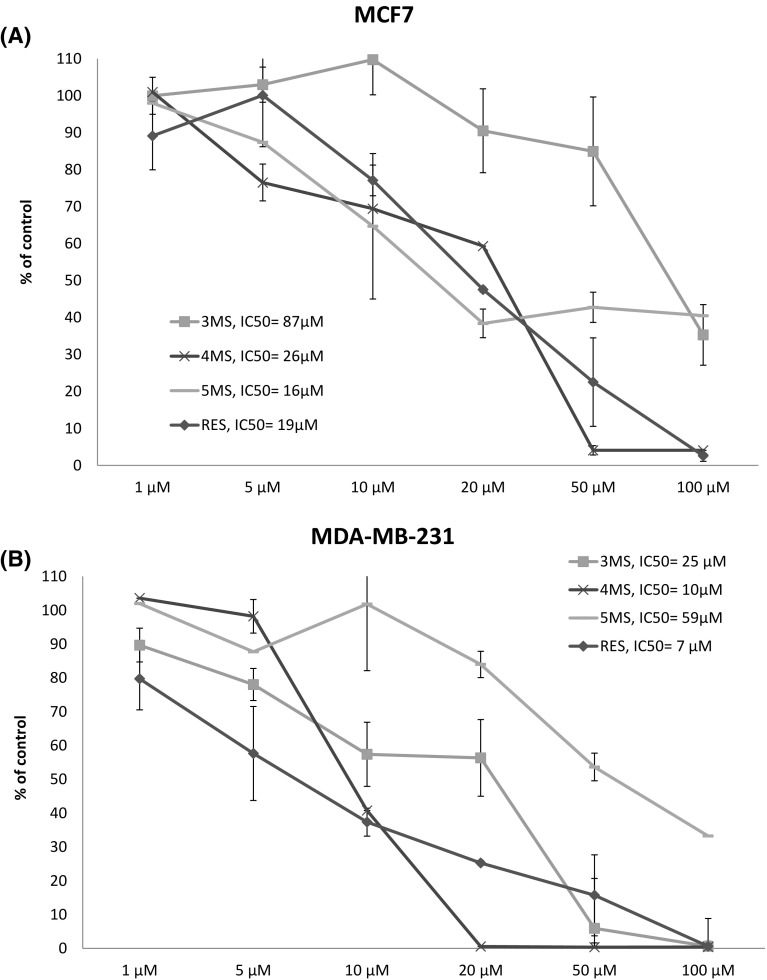
The effect of resveratrol (RES) and synthetic tri (3MS), tetra (4MS), penta (5MS) methoxy-stilbenes on the MCF7 (**a**) and MDA-MB-231 (**b**) cell lines viability. The mean values ± SEM from three independent experiments run in triplicate are presented

The treatment with resveratrol or its methoxy derivatives reduced the viability of both MCF7 and MDA-MB-231 cells, basically in a dose-dependent manner (Fig. 1). Generally, the methoxy-stilbenes showed lower cytotoxicity than resveratrol. 5MS showed the highest cytotoxicity in MCF7 cell with IC_50_ 16 µM, while 4MS in MDA-MB-231 (IC_50_ 10 µM). The 3MS in MCF7 cells and 5MS in MDA-MB-231 cells showed the lowest cytotoxic effect.

### Resveratrol and methoxy-stilbenes reduce the expression of ERα in MCF7cells

**Fig. 2:**
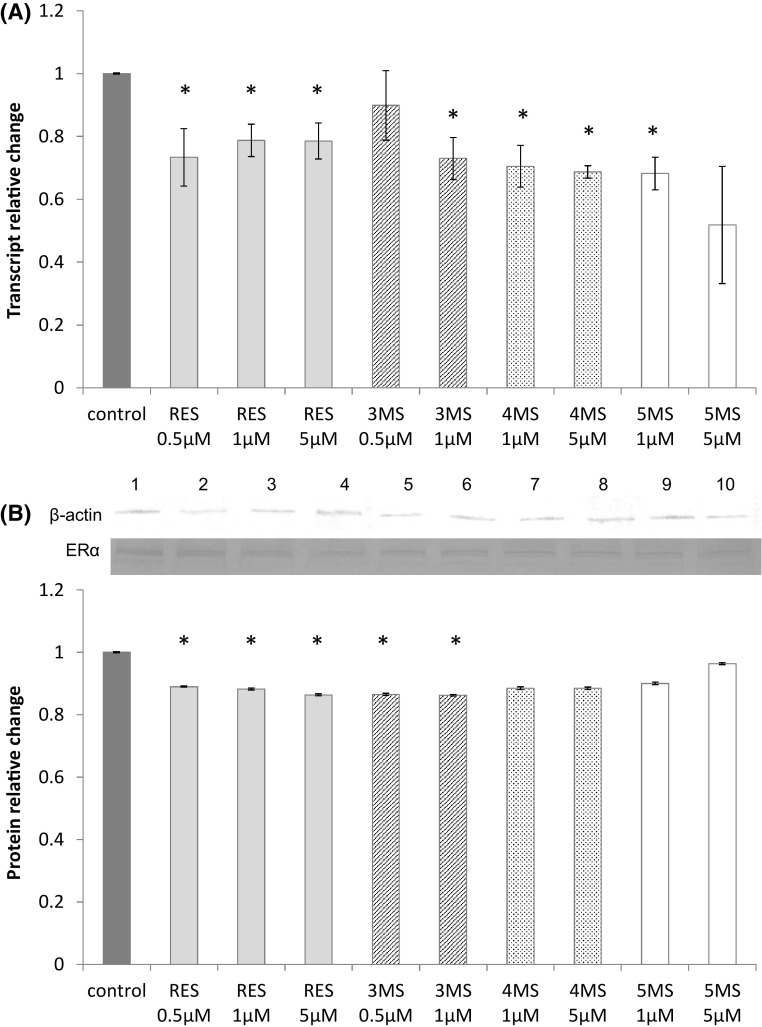
The effect of 72-h incubation with resveratrol (RES) and synthetic methoxy-*trans*-stilbenes on the level of the *ERα* transcript (**a**) and protein (**b**) in MCF7 cells. The values were calculated as a relative change in transcript or protein level in comparison to control cells (expression equals 1). The mean values ± SEM from three independent experiments run in triplicate are presented. *Mean values were significantly different from the control group (*P* < 0.05). Western blot analysis: representative blot is shown: (lane 1) control; (lanes 2, 3, 4) resveratrol; (lanes 5, 6) 3,4,2′-trimethoxy-*trans*-stilbene (3MS); (lanes 7, 8) 3,4,2′,4′-tetramethoxy-*trans*-stilbene (4MS); (lanes 9, 10) 3,4,2′,4′,6′-pentamethoxy-*trans*-stilbene (5MS)

Treatment of MCF7 cells with resveratrol for 72 h reduced the expression of *ERα* both on mRNA and protein levels (Fig. 2). Similar or even slightly higher effect was observed in the case of 3MS. The other methoxy-stilbenes tended to decrease mRNA transcripts but this effect was not confirmed on protein level.

### The effect of resveratrol and methoxy-stilbenes on the expression of CYP19

**Fig. 3:**
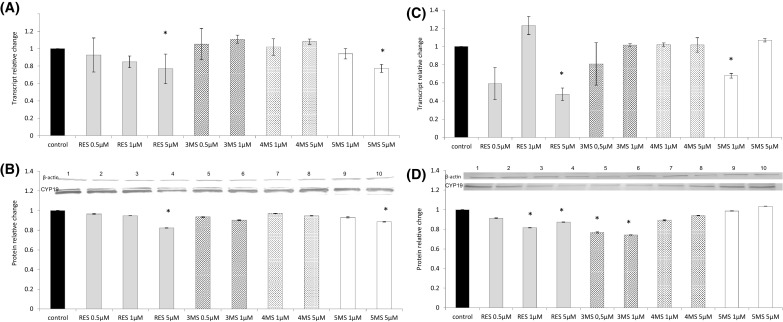
The effect of 72-h incubation with resveratrol (RES) and synthetic methoxy-*trans*-stilbenes on the level of the *CYP19* transcript (**a, c**) and protein (**b, d**) in MCF7 (**a, b**) and MDA-MB-231 (**c, d**) cells. The values were calculated as a relative change in transcript or protein level in comparison to control cells (expression equals 1). The mean values ± SEM from 3 independent experiments run in triplicate are presented. *Mean values were significantly different from the control group (*P* < 0.05). Western blot analysis: representative blot is shown: (lane 1) control; (lanes 2, 3, 4) resveratrol; (lanes 5, 6) 3,4,2′-trimethoxy-*trans*-stilbene (3MS); (lanes 7, 8) 3,4,2′,4′-tetramethoxy-*trans*-stilbene (4MS); (lanes 9, 10) 3,4,2′,4′,6′-pentamethoxy-*trans*-stilbene (5MS)

*CYP19* encodes aromatase, the key enzyme of estrogens biosynthesis. Treatment with stilbenes reduced the expression of *CYP19* in both cell lines. In MCF7 cells the most significant effect, but comparable to resveratrol in the dose of 5 µM, was exerted by 5MS. In MDA-MB-231 cells 3MS similarly as resveratrol in both tested doses significantly decreased CYP19 protein level (Fig. 3).

### 3MS reduces the activity of recombinant CYP19

In order to assess the effect of tested methoxy*-*stilbenes on aromatase activity, the recombinant CYP19 system was used. Basically, all these compounds did not affect significantly the activity of this enzyme. However, the 3MS was shown to be the most potent inhibitor with IC_50_ ~ 85 µM (Table 1).

**Table 1:** The effect of methoxy-stillbenes on recombinant aromatase activity in vitro

Compound	IC_50_^a^ (µM)
3MS	85
4MS	> 100
5MS	> 100

^a^IC_50_ values were determined using non-linear regression methods by GraphPad Prism software (San Diego, CA)

### 17β-estradiol (E2) level in culture medium

**Fig. 4:**
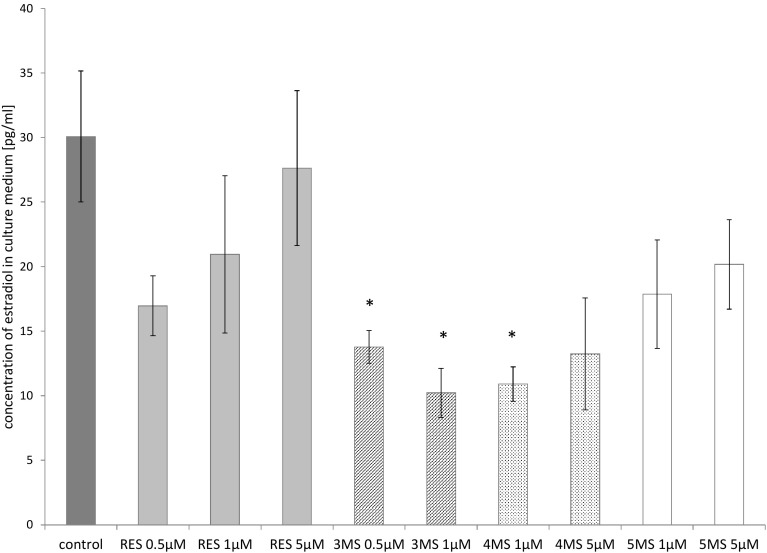
The effect of 72-h incubation with resveratrol (RES) and synthetic methoxy-*trans*-stilbenes on the level of 17β-estradiol in MCF7 cells measured in the culture medium by ELISA test. The level of 17β-estradiol in MDA-MB-231 cells was under the range of detection by the method (data not shown). The mean values ± SEM from three independent experiments run in triplicate are presented. *Mean values were significantly different from the control group (*P* < 0.05). 3,4,2′-trimethoxy-*trans*-stilbene (3MS); 3,4,2′,4′-tetramethoxy-*trans*-stilbene (4MS); 3,4,2′,4′,6′-pentamethoxy-*trans*-stilbene (5MS)

Treatment with stilbenes reduced the level of E2 in culture medium of MCF7 cells. The most pronounced effect was again in the case of 3MS which reduced the concentration of E2 in both tested doses, while 4MS–at the dose of 1 µM only (Fig. 4). In MDA-MB-231 cells, the estradiol concentrations were very low, and did not allow the calculation of statistical differences between untreated and treated cells with analyzed compounds (data not shown).

### The effect of resveratrol and methoxy-stilbenes on the expression of AhR, CYP1A1, and CYP1B1

**Fig. 5:**
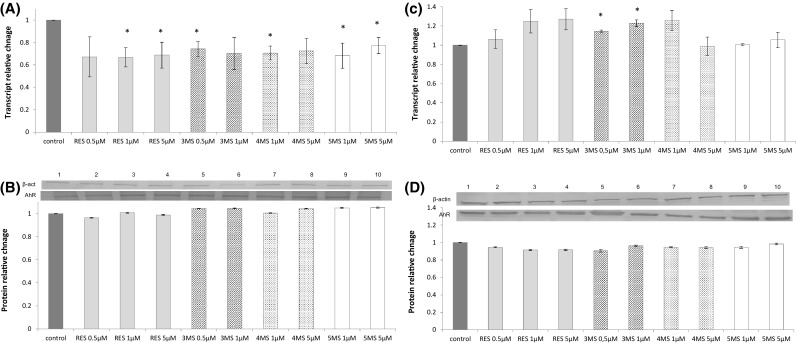
The effect of 72-h incubation with resveratrol (RES) and synthetic methoxy-*trans*-stilbenes on the level of the *AhR* transcript (**a, c**) and protein (**b, d**) in MCF7 (**a, b**) and MDA-MB-231 (**c, d**) cells. The values were calculated as a relative change in transcript or protein level in comparison to control cells (expression equals 1). The mean values ± SEM from three independent experiments run in triplicate are presented. *Mean values were significantly different from the control group (*P* < 0.05). Western blot analysis: representative blot is shown: (lane 1) control; (lanes 2, 3, 4) resveratrol; (lanes 5, 6) 3,4,2′-trimethoxy-*trans*-stilbene (3MS); (lanes 7, 8) 3,4,2′,4′-tetramethoxy-*trans*-stilbene (4MS); (lanes 9, 10) 3,4,2′,4′,6′-pentamethoxy-*trans*-stilbene (5MS)

**Fig. 6:**
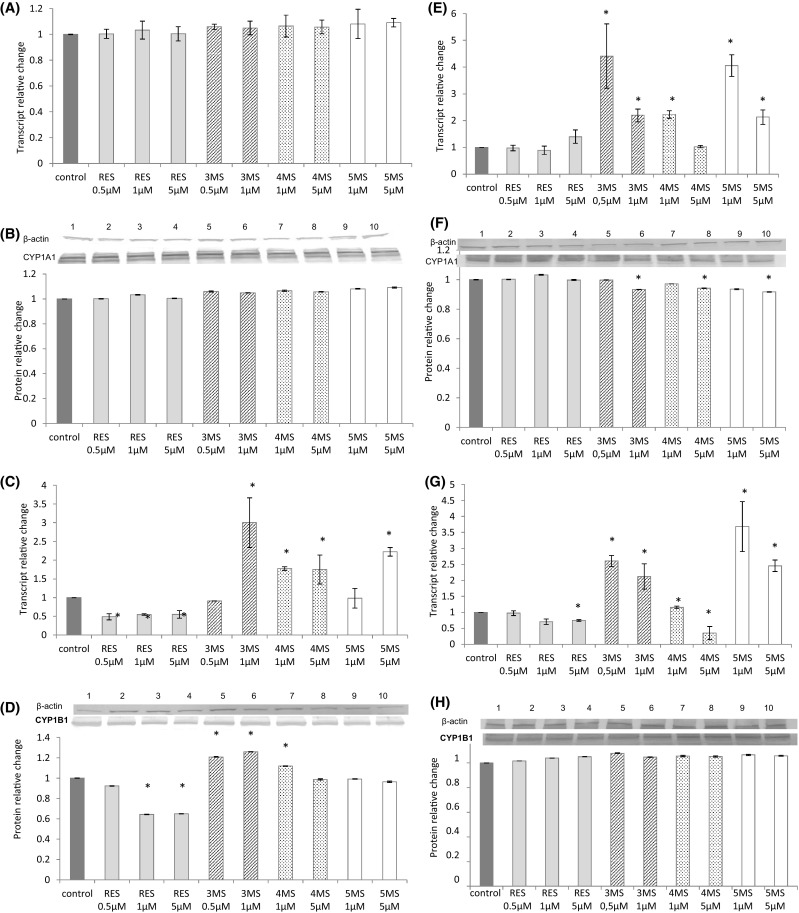
The effect of 72-h incubation with resveratrol (RES) and synthetic methoxy-*trans*-stilbenes on the level of the *CYP1A1* transcript (**a**) and protein (**b**), *CYP1B1* transcript (**c**) and protein (**d**) in MCF7 cells, *CYP1A1* transcript (**e**) and protein (**f**), *CYP1B1* transcript (**g**) and protein (**h**) in MDA-MB-231 cells. The values were calculated as a relative change in transcript or protein level in comparison to control cells (expression equals 1). The mean values ± SEM from three independent experiments run in triplicate are presented. *Mean values were significantly different from the control group (*P* < 0.05). Western blot analysis: representative blot is shown: (lane 1) control; (lanes 2, 3, 4) resveratrol; (lanes 5, 6) 3,4,2′-trimethoxy-*trans*-stilbene (3MS); (lanes 7, 8) 3,4,2′,4′-tetramethoxy-*trans*-stilbene (4MS); (lanes 9, 10) 3,4,2′,4′,6′-pentamethoxy-*trans*-stilbene (5MS)

Reduced expression of *AhR* was noticed as a result of treatment of MCF7 cells with all stilbenes, but only at the transcript level (Fig. 5). In contrast to MCF7 cells, in more aggressive MDA-MB-231 breast cancer cells, AhR transcript level showed tendency to augment particularly as a result of treatment with 3MS. Resveratrol reduced both transcript and protein levels of CYP1B1 in MCF7 cells, while methoxy-stilbenes tended to increase the expression of this cytochrome isoform. All tested stilbenes did not change the expression of CYP1A1 in MCF7 cells (Fig. 6a–d). In contrast in MDA-MB-231 methoxy-stilbenes at the higher doses reduced the CYP1A1 protein level, but transcript levels showed tendency to increase. CYP1B1 transcript was reduced as a result of treatment with resveratrol and 4MS at the dose of 5 µM. However, this effect was not confirmed at protein level (Fig. 6e–h).

## Discussion

The limitations of resveratrol such as unfavorable pharmacokinetics, extensive metabolism (glucuronide or sulfate conjugation) and low bioavailability [2] despite its promising health beneficial effects, prompt to design new stilbenes derivatives with similar or stronger biological activities, but possibly better pharmacokinetics. Implementation of methoxy instead of hydroxyl groups into stilbene rings may protect the core molecule against glucuronidation or sulphatation and seems to be good option. Moreover, methoxylation increases lipophilicity and may enhance cell membrane permeability [22].

This study focused on the evaluation of the effects of new synthetic methoxy-stilbenes with 3,4, and 5 methoxy groups in the positions 3,4,2′,4′,6′ of stilbene ring, respectively, on the expression of genes encoding proteins involved in estrogen signaling and metabolism in two breast cancer cell lines: benign MCF7 and metastatic (ER-negative) MDA-MB-231. For comparison with the data previously presented in MCF10 cells, the same doses and treatment protocol were applied [5].

The obtained results showed weak or moderate effects of these stilbenes on the evaluated parameters and in general, less significant than that observed in MCF10 cell line derived from spontaneously immortalized breast epithelial cells [18].

Generally the methoxy-stilbenes showed lower cytotoxicity than resveratrol, although this effect varied between both cell lines tested. In MCF7 cells the least cytotoxic was 3MS, while in MDA-MB-231 it was 5MS. Moreover, 3MS seemed to be more potent modulator of the expression of ERα than other methoxy-stilbenes in estrogen-dependent MCF7 cells. This derivative also reduced CYP19 protein level in MDA-MB-231. Moreover, 3MS significantly reduced the production of estradiol, as evaluated on the base of the measurement of its level in culture medium of MCF7 cells. However, in these cells similarly as in MCF10 cells, 5MS was more efficient inhibitor of *CYP19* expression than 3MS, but only at the transcript level.

This observation suggests that 3MS may affect the estrogens synthesis through down-regulation of *CYP19* expression more efficiently in cancer breast metastatic cells, while 5MS in benign cells.

The difference between mRNA transcript and the corresponding protein levels were observed also in our previous studies and may suggest that a certain threshold level of mRNA must be achieved before the protein can be translated, or cell-specific posttranscriptional modifications including proteolytic degradation can modulate protein levels [23, 24].

On the other hand, it is worth to note that 3MS also inhibited the most the aromatase activity in a recombinant protein system, although in comparison to resveratrol the IC_50_ value for 3MS was two times higher (IC_50_ value of ∼ 40 µM) as Wang and Leung showed in their study [25]. Thus, it is possible that this stilbene derivative affects *CYP19* gene expression and inhibits aromatase activity, but its effect on *CYP19* gene expression depends on cell type. The mechanism of this activity requires further studies.

It was shown recently that AhR (determined by *CYP1A1* expression at mRNA level) induces the mRNA expression of the aromatase gene in breast MCF7 cells [16].

The inhibition of AhR might be in turn responsible for aromatase gene down-regulation and reduced activity.

In our study, both resveratrol and the methoxy-stilbenes did not affect significantly the expression of, neither *AhR*, nor *CYP1A1*, at least at protein level. However, in contrast to resveratrol which reduced both transcript and protein levels of CYP1B1, methoxy-stilbenes showed tendency to increase CYP1B1 protein level in MCF7 cells. Thus, the reduced expression of *CYP19* by stilbenes tested in this study rather cannot be related to AhR inhibition.

The data on the effect of methoxy-stilbenes on the expression of genes encoding enzymes involved in estrogen metabolism are limited. Some of the stilbene methoxy derivatives were pointed out as potent inhibitors of CYP1 family of proteins. In this regard, 2,4,3′,5′-tetramethoxystilbene (TMS) was shown to be a strong modulator of CYP1B1 gene expression as well as potent selective inhibitor in vitro [26].

Additionally, more recent study showed that this *trans*-stilbene analog inhibits CYP1B1 activity, but does not reduce its mRNA level in vivo. However, the results of this investigation revealed also unexpected estrogen agonistic actions of TMS at high doses (50 mg/kg/day) [27]. The authors concluded that inhibitory properties of TMS, as well as the effects on developing breast, could implicate a role for TMS in breast cancer prevention, but only in low doses and on developing breast.

To certain extent the same interpretation may be applied to methoxy-stilbenes investigated in our current and previous studies. Our earlier data showed that 3,4,2′-trimethoxy-*trans*-stilbene was a potent inhibitor of human recombinant CYP1B1 activity [28]. The reduced expression of AhR, CYP1A1, and 1B1 was also found as a result of treatment with all these compounds in MCF10A cells, a model of the early stages of breast carcinogenesis.

It is possible that in more advanced stages of this process other *trans*-stilbenes modifications may be more efficient. In this regard, with respect to aromatase inhibition, the benzyl-substituted series was more potent than the methyl-substituted derivatives of resveratrol, and 3-*O*-benzyl resveratrol was about eightfold more active than resveratrol [29]. The moderate and non-selective aromatase inhibitory activity of resveratrol was improved about 100-fold by replacement of the ethylenic bridge with a thiadiazole and the phenyl rings with pyridines. The aromatase inhibitory activity was also enhanced over 6000-fold by using a 1,3-thiazole as the central ring and modifying the substituents on the ‘A’ ring to target the Met374 residue of aromatase [30].

In summary, the new methoxy-stilbenes might be potentially useful in prevention and/or reversal of the early stages of breast cancer development; however, more profound experimental studies are necessary in order to explain the mechanism of their biological activity.

## References

[1] Sirerol JA, Rodroguez ML, Mena S, Asensi MA, Estrela JM, Ortega AL (2016). Role of natural stilbenes in the prevention of cancer. Oxid Med Cell Longev.

[2] Cottari CH, Nivet-Antoine V, Beaudeux JL (2014). Review of recent data on the metabolism, biological effects, and toxicity of resveratrol in humans. Mol Nutr Food Res.

[3] Piotrowska H, Myszkowski K, Abraszek J, Kwiatkowska-Borowczyk E, Amarowicz R, Murias M, Wierzchowski M, Jodynis-Liebert J (2014). DMU-212 inhibits tumor growth in xenograft model of human ovarian cancer. Biomed Pharmacother.

[4] Piotrowska-Kempisty H, Klupczyńska A, Trzybulska D, Kulcenty K, Sulej-Suchomska AM, Kucińska M, Mikstacka R, Wierzchowski M, Murias M, Baer-Dubowska W, Kokot Z, Jodynis-Liebert J (2017). Role of CYP1A1 in the biological activity of methylated resveratrol analogue, 3,4,5,4′-tetramethoxystilbene (DMU-212) in ovarian cancer A-2780 and non-cancerous HOSE cells. Toxicol Lett.

[5] Licznerska B, Szaefer H, Wierzchowski M, Sobierajska H, Baer-Dubowska W (2017). Resveratrol and its methoxy derivatives modulate the expression of estrogen metabolism enzymes in breast epithelial cells by AhR down-regulation. Mol Cell Biochem.

[6] Cui J, Shen Y, Li R (2013). Estrogen synthesis and signaling pathways during ageing: from periphery to brain. Trends Mol Med.

[7] Jia M, Dahlman-Wright K, Gustafsson JA (2015). Estrogen receptor alpha and beta in health and disease. Best Pract Res Clin Endocrinol Metab.

[8] Huang B, Omoto Y, Iwase H, Yamashita H, Toyama T, Coombes RC, Filipovic A, Warner M, Gustafsson J (2014). Differential expression of estrogen receptor alpha, beta1, and beta2 in lobular and ductal breast cancer. Proc Natl Acad Sci USA.

[9] Couse JF, Korach KS (1999). Estrogen receptor null mice: what have we learned and where will they lead us?. Endocr Rev.

[10] Yasuda MT, Sakakibara H, Shimoi K (2017). Estrogen- and stress-induced DNA damage in breast cancer and chemoprevention with dietary flavonoid. Genes Environ.

[11] Ohtake F, Takeyama K, Matsumoto T, Kitagawa H, Yamamoto Y, Nohara K, Tohyama C, Krust A, Mimura J, Chambon P, Yanagisawa J, Kato S, Fujii Kuriyama Y (2003). Modulation of oestrogen receptor signalling by association with the activated dioxin receptor. Nature.

[12] Wormke M, Stoner M, Saville B, Walker K, Abdelrahim M, Burghardt R, Safe S (2003). The aryl hydrocarbon receptor mediates degradation of estrogen receptor alpha through activation of proteasomes. Mol Cell Biol.

[13] Zhao S, Kanno Y, Nakayama M, Makimura M, Ohara S, Inouye Y (2012). Activation of the aryl hydrocarbon receptor represses mammosphere formation in MCF-7 cells. Cancer Lett.

[14] Chan MY, Huang H, Leung LK (2010). 2,3,7,8-Tetrachlorodibenzo-para-dioxin increases aromatase (CYP19) mRNA stability in MCF-7 cells. Mol Cell Endocrinol.

[15] Cheshenko K, Brion F, Le Page Y, Hinfray N, Pakdel F, Kah O, Segner H, Eggen RI (2007). Expression of zebra fish aromatase cyp19a and cyp19b genes in response to the ligands of estrogen receptor and aryl hydrocarbon receptor. Toxicol Sci.

[16] Saito R, Miki Y, Hata S, Ishida T, Suzuki T, Ohuchi N, Sasano H (2017). Aryl hydrocarbon receptor induced intratumoral aromatase in breast cancer. Breast Cancer Res Treat.

[17] Casper RF, Quesne M, Rogers IM, Shirota T, Jolivet A, Milgrom E, Savouret JF (1999) Resveratrol has antagonist activity on the aryl hydrocarbon receptor: implications for prevention of dioxin toxicity. Mol Pharmacol 56:784–790. http://molpharm.aspetjournals.org/content/56/4/784.long 10496962

[18] Soule HD, Maloney TM, Wolman SR, Peterson WD, Brenz R, McGrath CM, Russo J, Pauley RJ, Jones RF, Brooks SC (1990). Isolation and characterization of a spontaneously immortalized human breast epithelial cell line, MCF-10. Cancer Res.

[19] Russo J, Fernandez SV, Russo PA, Fernbaugh R, Sheriff FS, Lareef HM, Garberand J, Russo JH (2006). 17-b-Estradiol induces transformation and tumorigenesis in human breast epithelial cells. FASEB J.

[20] Szaefer H, Krajka-Kuźniak V, Licznerska B, Bartoszek A, Baer-Dubowska W (2015). Cabbage juices and indoles modulate the expression profile of AhR, ERα, and Nrf2 in human breast cell lines. Nutr Cancer.

[21] Jin RY, Gui WJ, Guo YR, Wang CM, Wu JX, Zhu GN (2008). Comparison of monoclonal antibody-based ELISA for triazophos between the indirect and direct formats. Food Agric Immunol.

[22] Lin H-S, Ho PC (2009). A rapid HPLC method for the quantification of 3,5,4′-trimethoxy-trans-stilbene (TMS) in rat plasma and its application in pharmacokinetics study. J Pharm Biomed Anal.

[23] McFadyen MCE, Rooney PH, Melvin WT, Murray GI (2003). Quantitative analysis of the Ah receptor/cytochrome P450 CYP1B1/CYP1A1 signalling pathway. Biochem Pharmacol.

[24] Bandiera S, Weidlich S, Harth V, Broede P, Ko J, Friedberg T (2005). Proteasomal degradation of human cytochrome P450 1B1 (CYP1B1): effect of the Asn^453^Ser polymorphism on the post-translational regulation of CYP1B1 expression. Mol Pharmacol.

[25] Wang Y, Leung LK (2007). Pharmacological concentration of resveratrol suppresses aromatase in JEG-3 cells. Toxicol Lett.

[26] Chun Y-J, Lee S-K, Kim MY (2005). Modulation of human cytochrome P450 1B1 expression by 2,4,3′,5′-tetramethoxystilbene. Drug Metab Dispos.

[27] Kim T, Park H, Yue W, Wang J-P, Atkins KA, Zhang Z, Rogan EG, Cavalieri EL, Mohammad KS, Kim S, Santen RJ, Aiyar SE (2011). Tetra-methoxystilbene modulates ductal growth of the developing murine mammary gland. Breast Cancer Res Treat.

[28] Mikstacka R, Wierzchowski M, Dutkiewicz Z, Gielara-Korzańska A, Korzański A, Teubert A, Sobiak S, Baer-Dubowska W (2014). 3,4,20-Trimethoxy-trans-stilbene: a potent CYP1B1 inhibitor. Med Chem Commun.

[29] Orsini F, Verotta L, Klimo K, Gerhäuser C (2016). Synthesis of resveratrol derivatives and in vitro screening for potential cancer chemopreventive activities. Arch Pharm.

[30] Mayhoub AS, Marler L, Kondratyuk TP, Park EJ, Pezzuto JM, Cushman M (2012). Optimization of the aromatase inhibitory activities of pyridylthiazole analogues of resveratrol. Bioorg Med Chem.

